# 1409. Pulmonary Non-tuberculous *Mycobacterium* Infection (PNTMI) and COVID-19: Characterization of the National COVID Collaborative Cohort (N3C)

**DOI:** 10.1093/ofid/ofab466.1601

**Published:** 2021-12-04

**Authors:** Carlos E Figueroa Castro, William Hersh

**Affiliations:** 1 Medical College of Wisconsin, Milwaukee, Wisconsin; 2 Oregon Health & Science University, Portland, Oregon

## Abstract

**Background:**

Establishing whether a low-prevalence clinical condition is a risk factor for COVID-19 infection, or serious adverse outcomes, is difficult due to a limited number of patients, and lack of access to patient’s data by researchers. The National COVID Collaborative Cohort (N3C), a centralized national data resource to study COVID-19, provides access to structured clinical data derived from electronic health records. As of June 2021, N3C contains data on 6,193,738 patients (2,090,138 with COVID-19, 33.7%) from 55 participating sites (Figure 1). We describe the characteristics of patients with PNTMI based on COVID-19 infection status.

Figure 1

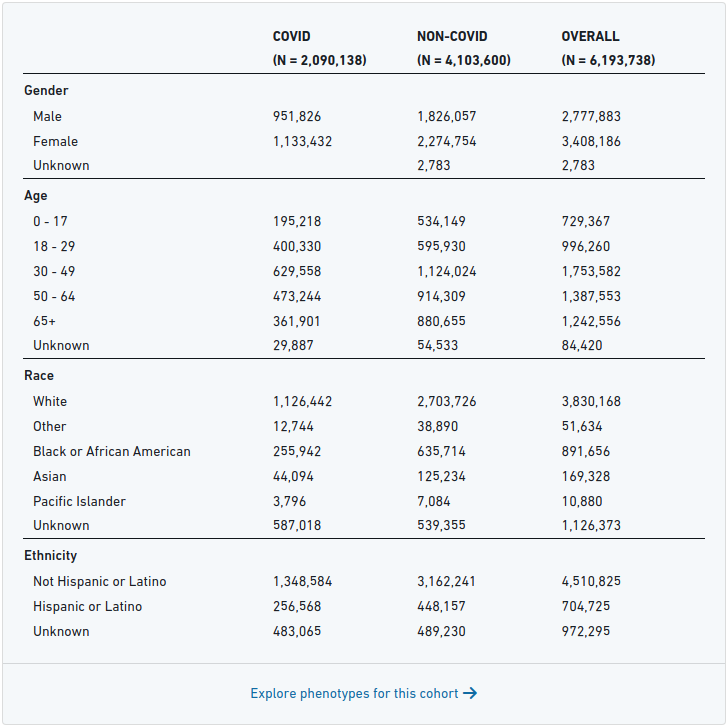

N3C Basic Demographic Data

**Methods:**

COVID-19 is defined by positive lab result (PCR, antigen, or antibody) or COVID-19 coding diagnosis, as defined by N3C. PNTMI phenotype was built with N3C Data Enclave concept set tool, and ATLAS (https://atlas.ohdsi.org/). We limited analysis to adults (18 years-old or older). We used de-identified data sets stripped of protected health information (PHI). We used N3C Data Enclave analytical tools for exploratory data analysis, and descriptive statistics.

**Results:**

We identified five hundred and eighty six individuals from 19 sites fulfilling the PNTMI phenotype (9.46 cases per 100,000 people). After our age limit, 555 individuals were included for analysis (Figure 2). 340 were females (61.3%), 447 of white race (80.5%), and 30 were Hispanic (5.4%). Additional descriptive statistics and statistical significance testing are provided (Table 1). The most common concept were "Non-tuberculous mycobacterial pneumonia", and "Pulmonary *Mycobacterium avium* complex infection". Four sites accounted for more than 50% of identified patients (Figure 2). We identified 24 individuals with COVID-19 (4.32%), and 44 deaths in this cohort (7.9%). Deaths were unrelated to COVID-19 event.

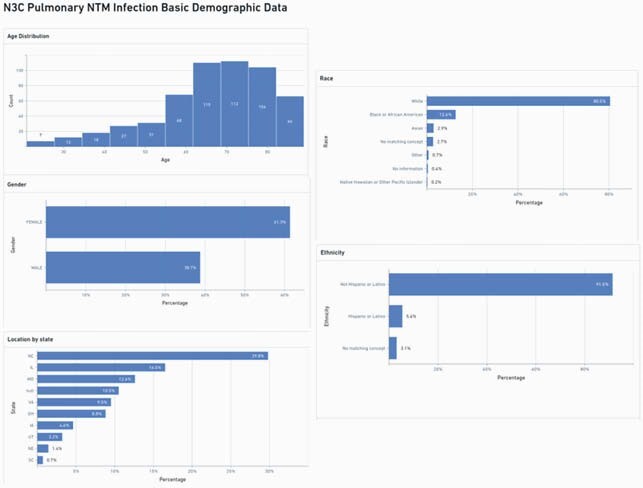

Figure 2. Basic demographic data of pulmonary non-tuberculous Mycobacterium infection phenotype in N3C

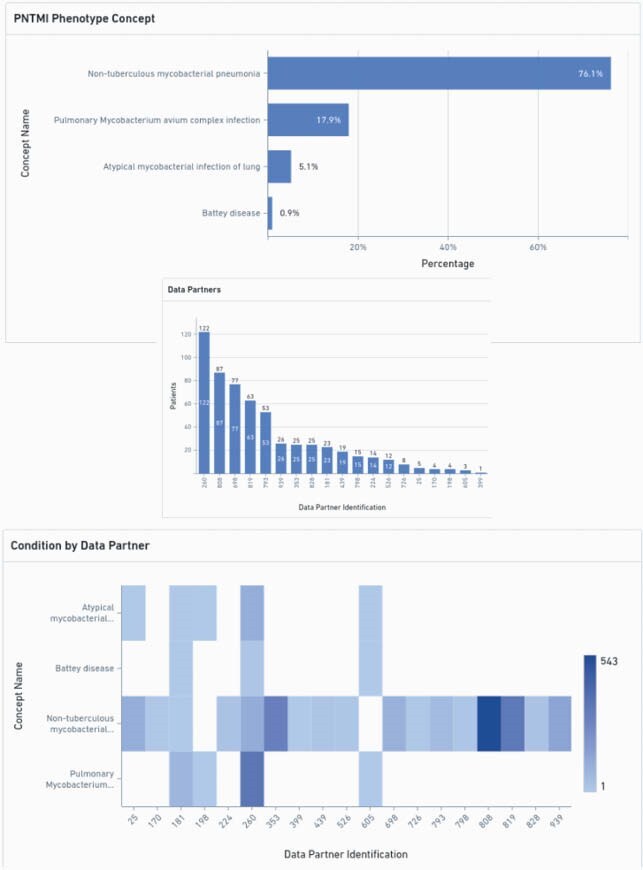

Figure 3. Concepts and data sources of pulmonary non-tuberculous Mycobacterium infection phenotype in N3C

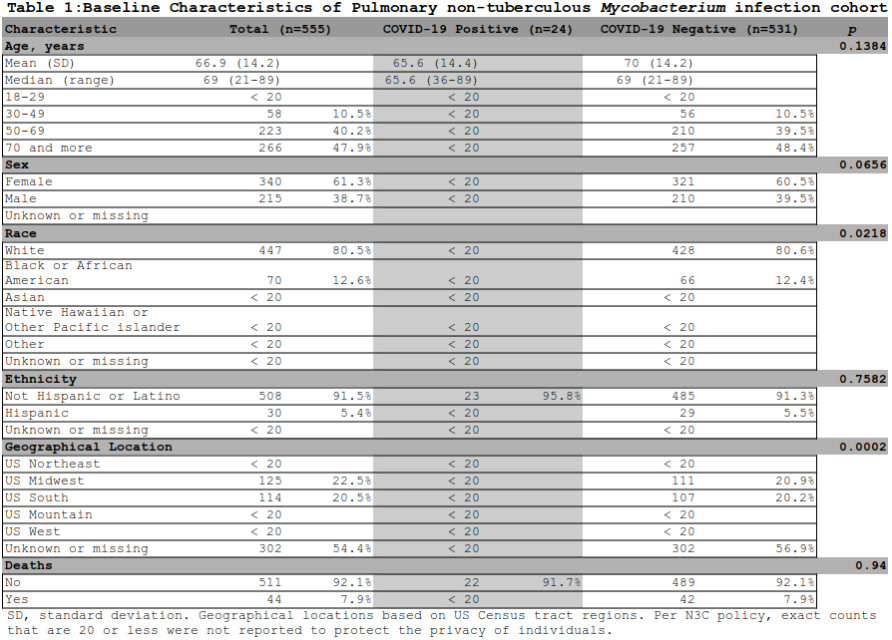

**Conclusion:**

In N3C, the PNTMI cohort has a lower proportion of COVID-19 infection than the general population, and it was not a cause of mortality. Further analysis to study impact of comorbidities, and differences in race and geographical location are warranted. N3C is a powerful research platform to study the impact of COVID-19 in special populations with low prevalence, and it can be used to study other populations of interest.

**Disclosures:**

**All Authors**: No reported disclosures

